# An X-ray gas monitor for free-electron lasers^1^

**DOI:** 10.1107/S1600577519005174

**Published:** 2019-06-12

**Authors:** Andrey A. Sorokin, Yilmaz Bican, Susanne Bonfigt, Maciej Brachmanski, Markus Braune, Ulf Fini Jastrow, Alexander Gottwald, Hendrik Kaser, Mathias Richter, Kai Tiedtke

**Affiliations:** a Deutsches Elektronen-Synchrotron (DESY), Notkestrasse 85, D-22607 Hamburg, Germany; b Ioffe Physico-Technical Institute, Polytekhnicheskaya 26, 194021 St Petersburg, Russian Federation; c Physikalisch-Technische Bundesanstalt (PTB), Abbestrasse 2–12, D-10587 Berlin, Germany

**Keywords:** free-electron lasers, vacuum ultraviolet, soft X-rays, hard X-rays, photon diagnostics

## Abstract

A novel X-ray gas monitor (XGM) has been developed which allows the measurement of absolute photon pulse energy and photon beam position at all existing and upcoming free-electron lasers (FELs) over a broad spectral range covering vacuum ultraviolet (VUV), extreme ultraviolet (EUV) and soft and hard X-rays. The XGM covers a wide dynamic range from spontaneous undulator radiation to FEL radiation and provides a temporal resolution of better than 200 ns. The XGM consists of two X-ray gas-monitor detectors (XGMDs) and two huge-aperture open electron multipliers (HAMPs). The HAMP enhances the detection efficiency of the XGM for low-intensity radiation down to 10^5^ photons per pulse and for FEL radiation in the hard X-ray spectral range, while the XGMD operates in higher-intensity regimes. The relative standard uncertainty for measurements of the absolute photon pulse energy is well below 10%, and down to 1% for measurements of relative pulse-to-pulse intensity on pulses with more than 10^10^ photons per pulse. The accuracy of beam-position monitoring in the vertical and horizontal directions is of the order of 10 µm.

## Abstract 2

Characterization of the absolute photon pulse energy and beam position of free-electron lasers (FELs) is essential for many user experiments, as well as for machine operators. Described here is an X-ray gas monitor which is a suitable tool for FEL photon diagnostics over a broad spectral range from vacuum ultraviolet to hard X-rays.

## 1. Introduction   

Characterization of free-electron laser (FEL) beam parameters such as the absolute photon flux is extremely important and a fundamental quantity for many user experiments, as well as for machine operators. State-of-the-art FEL facilities like FLASH, FERMI, LCLS, SACLA, SwissFEL, European XFEL and PAL-XFEL which are currently in operation generate highly intense and extremely short femtosecond photon pulses with a peak power of more than 10 GW in the spectral range from vacuum ultraviolet (VUV) to hard X-rays, and with a repetition rate of up to 4.5 MHz in the case of the European XFEL. Moreover, these facilities, except FERMI, are based on self-amplified spontaneous emission (SASE) and generate FEL pulses which have a chaotic nature regarding the statistical intensity fluctuation. This necessarily requires online non-invasive monitoring of the absolute pulse energy on a shot-to-shot basis with a sufficiently high temporal resolution.

At synchrotron radiation sources, semiconductor photodiodes of different types are widely used as transfer detector standards for measuring the absolute photon intensity in the spectral range from VUV to hard X-rays. Before use, these diodes are typically calibrated against electrical substitution radiometers established as primary detector standards in national metrological institutes such as the Physikalisch-Technische Bundesanstalt (PTB) in Germany (Krumrey & Ulm, 2001 ▸; Scholze *et al.*, 2003 ▸; Gottwald *et al.*, 2006 ▸, 2010 ▸), the National Institute of Advanced Industrial Science and Technology (AIST) in Japan (Morishita *et al.*, 2005 ▸; Kato *et al.*, 2007 ▸) and the National Institute of Standards and Technology (NIST) in the USA (Shaw *et al.*, 1999 ▸; Li *et al.*, 2006 ▸). The radiometers enable the measurement of radiant power with the highest relative standard uncertainties well below 1%, as validated, for example, by intercomparisons between PTB and AIST (Tanaka *et al.*, 2012 ▸) or between PTB and NIST (Gottwald *et al.*, 2011 ▸) for detectors in the VUV and X-ray range. However, the utilization of these detectors at FEL sources is restricted as the powerful FEL radiation can easily saturate or even destroy any semiconductor photodiodes. The radiometers, which are more stable under intense FEL radiation, are suitable for measuring the absolute FEL radiant power (Kato *et al.*, 2009 ▸, 2010 ▸, 2012 ▸; Saito *et al.*, 2010 ▸; Tanaka *et al.*, 2011 ▸, 2015 ▸, 2017 ▸). However, these techniques intercept the photon beam and so cannot be applied for online photon diagnostics, and they generally lack the required temporal resolution.

Gas detectors based on atomic photoionization, such as double ionization chambers (Samson, 1964 ▸; Samson & Haddad, 1974 ▸; Saito & Suzuki, 1998 ▸, 1999 ▸), are free of radiation-induced degradation and destruction. Nevertheless, their utilization at X-ray FELs is complicated. Double ionization chambers are operated at a high gas pressure in the range from 10^−1^ to 10^3^ Pa and thus absorb the photon beam significantly. Therefore, these devices are not suitable for online intensity monitoring either.

At FLASH, which was the first soft X-ray FEL (XFEL) in the world, gas-monitor detectors (GMDs) have been installed as a permanent part of the photon diagnostics system since its first day of operation in 2005 (Richter *et al.*, 2003 ▸; Sorokin *et al.*, 2004 ▸; Tiedtke *et al.*, 2008 ▸). The GMD represents a transfer detector standard calibrated against the PTB cryogenic radiometer (as primary standard) enabling non-invasive FEL pulse energy and photon beam position monitoring in the spectral range from VUV to soft X-rays. At FLASH, a set of four GMDs are placed behind the undulator line to assist machine set up, beam tuning, monitoring of the absolute FEL intensity and pointing of the photon beam (Tiedtke *et al.*, 2008 ▸). To characterize the FEL radiation at the experimental stations and behind the distributing and focusing mirrors, an upgraded version of the GMD, a so-called Round-Robin GMD (RRGMD), has been designed. The RRGMD is a compact and portable detector with an extended dynamic range for the extreme ultraviolet (EUV) and soft X-ray range which can easily be moved to different FLASH beamlines or to other FELs around the world. The RRGMD was successfully tested at the SPring-8 EUV FEL (Saito *et al.*, 2010 ▸; Kato *et al.*, 2010 ▸) and LCLS (Tiedtke *et al.*, 2014 ▸) for measuring radiant power in the soft X-ray spectral range. At the LCLS facility, an RRGMD has recently been integrated in the Soft X-ray Research (SXR) instrument as a permanent part of the photon diagnostics system (Moeller *et al.*, 2015 ▸). The RRGMD has also been used to characterize the absolute VUV radiation from a high-order harmonics source (Leitner *et al.*, 2011 ▸). However, use of the GMD and RRGMD for monitoring an attenuated FEL beam is limited, in particular in the hard X-ray spectral range, due to the low photoionization cross sections of the detector gases which are several orders of magnitude lower here than in the VUV and soft X-ray regime (Henke *et al.*, 1993 ▸).

To overcome these challenges we have designed and constructed a new version of the gas detector, the XGM (X-ray gas monitor), based on previous experience. It consists of two X-ray gas-monitor detectors (XGMDs) and two huge-aperture open electron multipliers (HAMPs) and can be used in the broader spectral range from VUV to hard X-rays due to its higher detection efficiency. Moreover, an improved temporal resolution well below 200 ns is provided, fulfilling the demands of the state-of-the-art high-repetition-rate XFELs. For instance, the European XFEL operates in burst mode with a frequency of 4.5 MHz, *i.e.* with a separation between two subsequent pulses of just 220 ns. During its preparatory phase, the XGMD was already successfully tested in the photon energy range from 4.4 to 13.8 keV at the SACLA hard X-ray FEL (Kato *et al.*, 2012 ▸). The agreement between data obtained by the XGMD and the AIST cryogenic radiometer (within 4%) is well below their combined relative standard uncertainty, validating their capabilities. The XGMD was also used at the LCLS to characterize the X-ray Pump–Probe Instrument in the hard X-ray regime (Song *et al.*, 2019 ▸).

By 2017, 20 (identical) XGMDs and 14 HAMPs had been constructed for application at FLASH 2, the European XFEL, the SwissFEL, LCLS II and the EUCALL consortium. In 2017 the respective detectors were installed at FLASH 2 (Faatz *et al.*, 2016 ▸), the SwissFEL (Juranić *et al.*, 2018 ▸) and the European XFEL (Grünert *et al.*, 2019 ▸; Maltezopoulos *et al.*, 2019 ▸) to provide a permanent service to experimentalists and machine operators. In the near future, it is intended that the XGMD will also be implemented as a permanent part of the online photon diagnostics tool at the new facility LCLS II, which will provide X-rays in quasi-continuous-wave (quasi-cw) mode with a repetition rate of up to 100 kHz.

In the following sections, we describe the main principles of operation of the XGM, XGMD and HAMP, and present results of test and calibration measurements which were performed at the Metrology Light Source (MLS) of the Physikalisch-Technische Bundesanstalt (PTB) (Gottwald *et al.*, 2012 ▸, 2019 ▸) and at FLASH 2.

## 2. X-ray gas monitor (XGM)   

**Figure 1:**
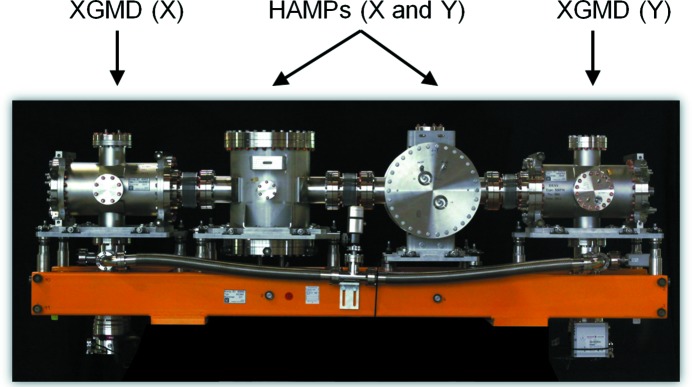
A picture of the XGM device. The left and right XGMDs are used for absolute average and pulse-resolved intensity, as well as for beam-position monitoring. The two HAMPs in the middle measure the relative pulse-resolved intensity and beam position with the help of in-house-made open electron multipliers.

A picture of an XGM, consisting of two XGMDs and two HAMPs mounted on common girder, is shown in Fig. 1 ▸. The total length of the XGM along the beam is 2 m and its mass is 600 kg. The operation of the XGMDs requires at least 10^10^ photons per pulse and provides reliable information about the absolute pulse energy with a temporal resolution of better than 10 ns. The HAMP detectors enhance the detection efficiency for low-intensity radiation down to 10^5^ photons per pulse as well as for the hard X-ray regime, and measure the pulse energy with a temporal resolution of better than 200 ns. In order to measure the beam position as well, in both horizontal and vertical directions, the two XGMDs and two HAMPs are equipped with split detection electrodes and are rotated by 90° to each other, respectively. Operation of the XGM is based on atomic photoionization of rare gases at relatively low pressures in the range 10^−4^ to 10^−2^ Pa.

### 2.1. X-ray gas-monitor detector (XGMD)   

The basic principle of the XGMD is that ions and photoelectrons created upon photoionization are simultaneously detected by simple metal plates. Hence, the detector is not only radiation-hard and transparent but also does not suffer from any kind of degradation.

**Figure 2:**
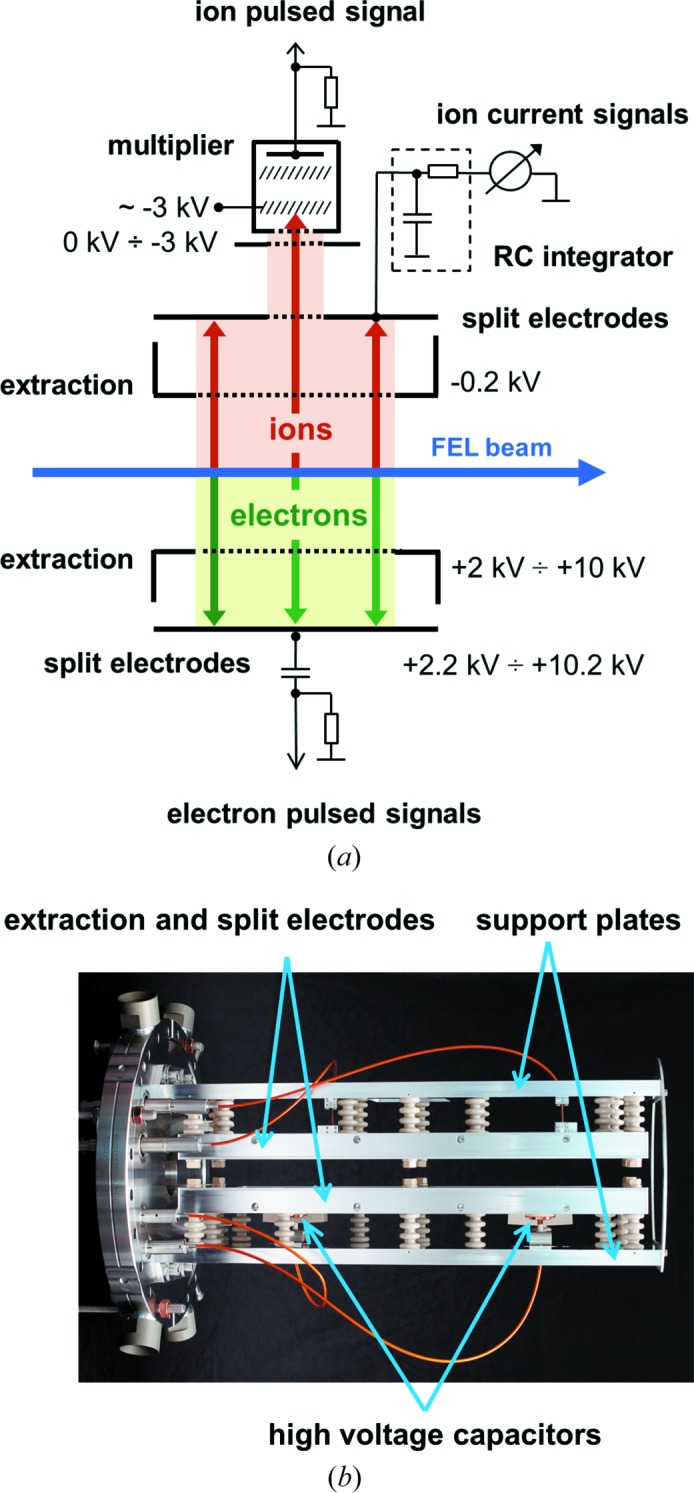
(*a*) A schematic diagram of the XGMD. (*b*) A picture of the XGMD (the commercial multiplier is not shown).

A general overview of the assembly and basic operation principles of the XGMD are shown in Fig. 2 ▸. The XGMD represents an ionization chamber consisting of a system of aluminium electrodes mounted on two supporting grounded plates attached to a standard DN200CF stainless steel flange. This flange is part of a vacuum chamber equipped with DN40CF entrance and exit flanges. The chamber is evacuated by a turbo-molecular pump to a residual pressure of less than 10^−5^ Pa. The target rare gas is introduced via a needle valve, homogeneously filling the chamber. A homogeneous pressure distribution is achieved by installing the needle valve between the pump and the vacuum chamber. With this design, the target gas atoms enter the vacuum chamber mainly by diffusion, leading to a homogeneous distribution within the interaction region. A confirmation of this can be found in the independence of the ion signal from the position of the photon beam in the interaction region of the XGMD (see Fig. 7). An additional proof is the good agreement between the XGMD and the radiometer when measuring pulse energy, as mentioned in the *Introduction* [Sec sec1]. A system of differential pumping units is used to separate the ultra-high-vacuum beamline from the device. As mentioned above, the target gas pressure is below 10^−2^ Pa. In this regime, the operation of the XGMD is not affected by any secondary effects upon photoionization, such as ionization by secondary electrons released from the target gas atoms or charge exchange between ions and atoms.

**Figure 3:**
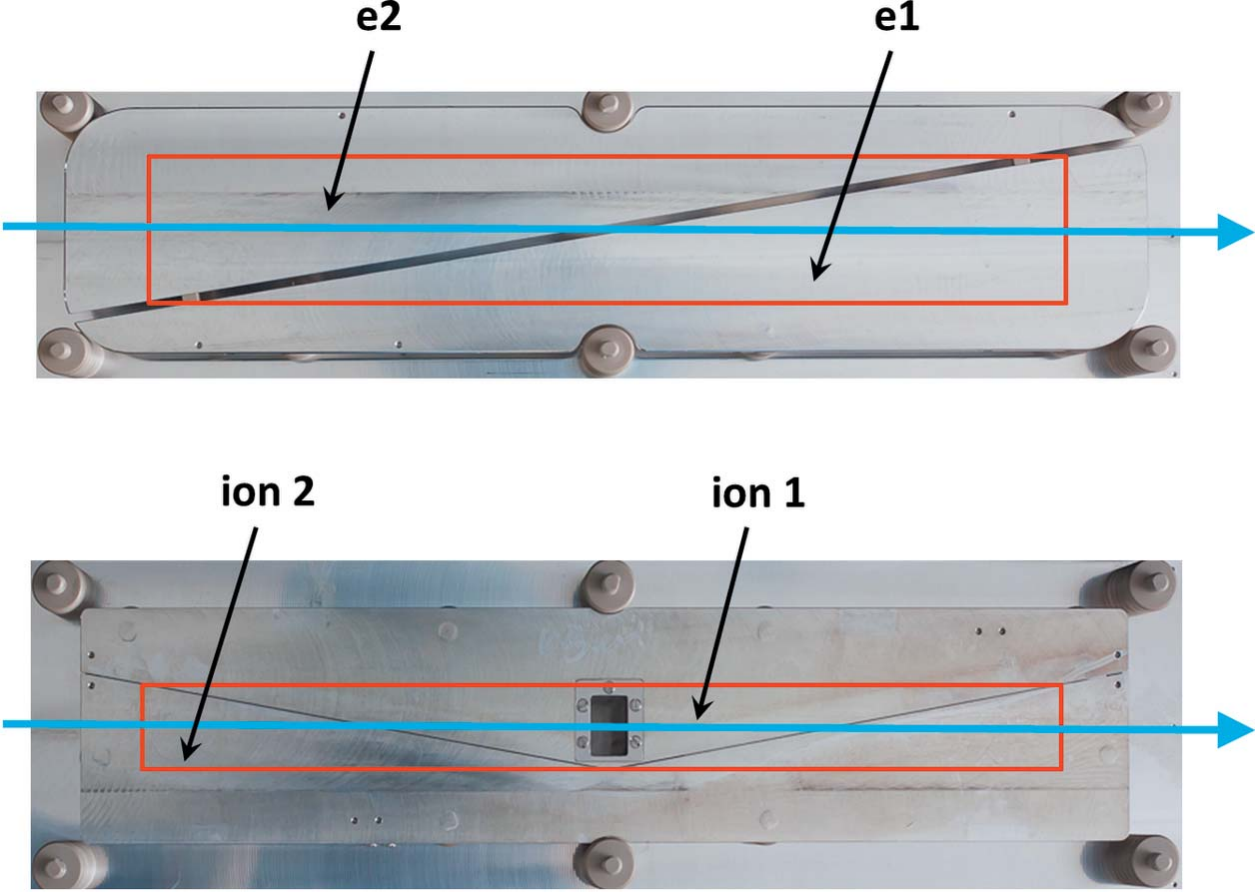
(Top) A top view of a triangular split electrode for electron detection. (Bottom) A top view of a linear split electrode for ion detection, with the aperture in the central part which enables the transmission of a fraction of the ions towards the commercial open electron multiplier. Blue arrows represent the direction of the FEL beam. Red rectangles indicate the sensitive area in the respective extraction electrode which can be hit by charged particles, defined by the rectangular aperture.

The monochromatic photon beam enters the XGMD chamber and passes between two parallel extraction electrodes of 365 mm in length, separated by 22 mm. The created ions and photoelectrons are extracted and accelerated from the interaction volume in opposite directions by a homogeneous electric field. The static extraction field from 1 × 10^3^ V cm^−1^ to 5 × 10^3^ V cm^−1^ is high enough to ensure the complete separation of ions and photoelectrons. The choice of the extraction voltage is dependent on the photon energy and photon beam polarization, and thus on the kinetic energy and angular distribution of the photoelectrons liberated from the target atom. To check if the extraction field is high enough to separate the photoelectrons and ions in the interaction region, one can vary the extraction field in one XGMD of the XGM, keeping constant all electric potentials in the other XGMD as a reference, until the same measured ion signal is obtained in both devices. We obtain independence of the ion signal already at 2 × 10^3^ V cm^−1^ at a photon energy of 10 keV with xenon as the target gas. A large fraction of the charged particles pass through the rectangular apertures in the respective extraction electrodes, covered by Ni grids with 80% transparency, and are detected by the split electrodes shown in Fig. 3 ▸. The apertures are 290 mm in length along the beam path and 50 mm wide, defining the active area of the detection electrodes hit by charged particles. Thus, compared with early versions of the gas-monitor detectors with a length of only 30 mm, the detection efficiency of the XGMD is about ten times higher. The length is chosen in such a way that in the X-ray regime at least 10^4^ charged particles can be detected. Thus, 1% photoionization statistics are achieved, as determined by the Poission statistics according to 1/(10^4^)^1/2^. The detection of charged particles by simple metal electrodes guarantees a linear signal response, even for a large number of secondary particles of up to 10^10^ which might be created during a single FEL pulse.

**Figure 4:**
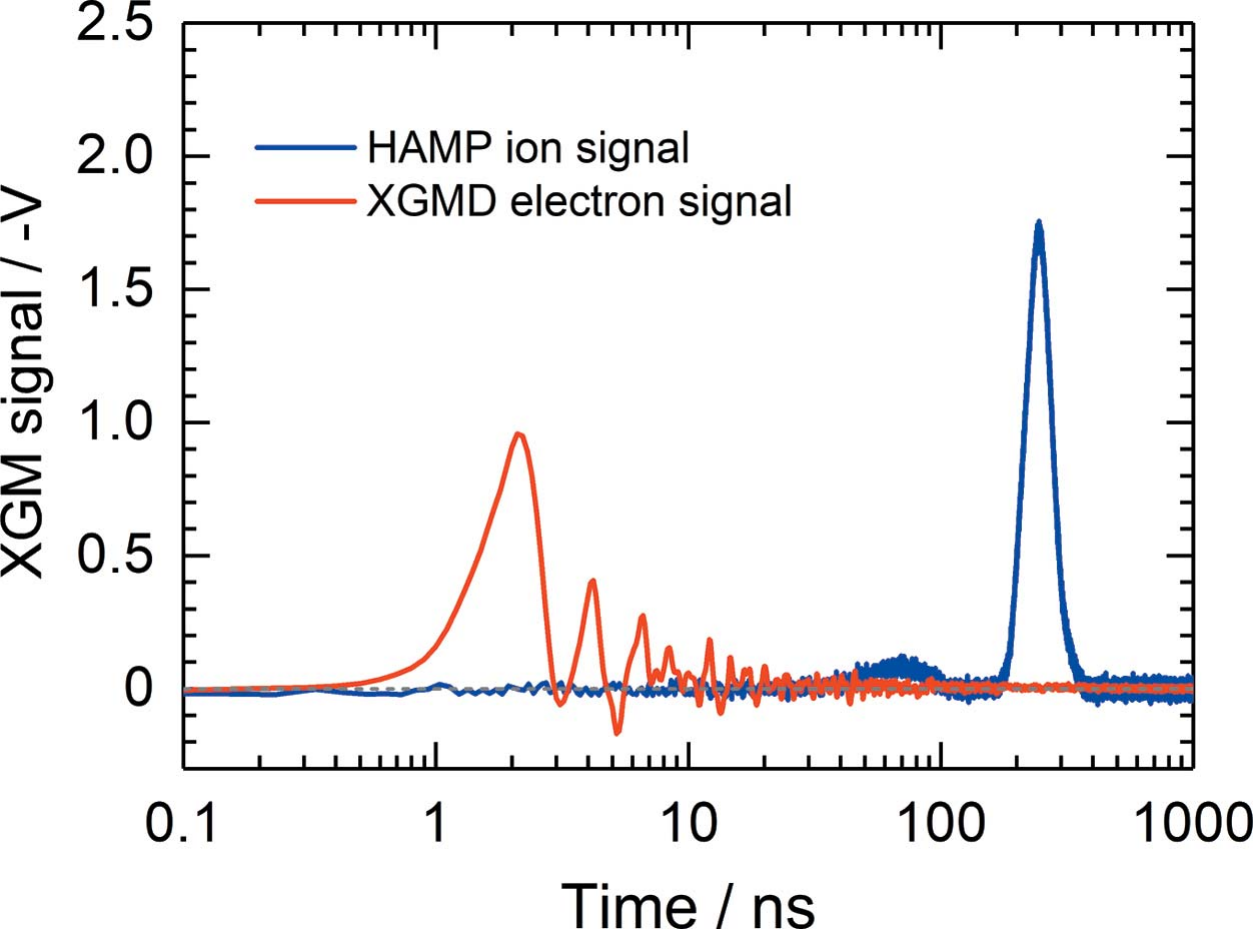
Typical pulse-resolved signals from the XGM measured with xenon. The data were obtained at FLASH2 at a wavelength of 13.5 nm.

**Figure 5:**
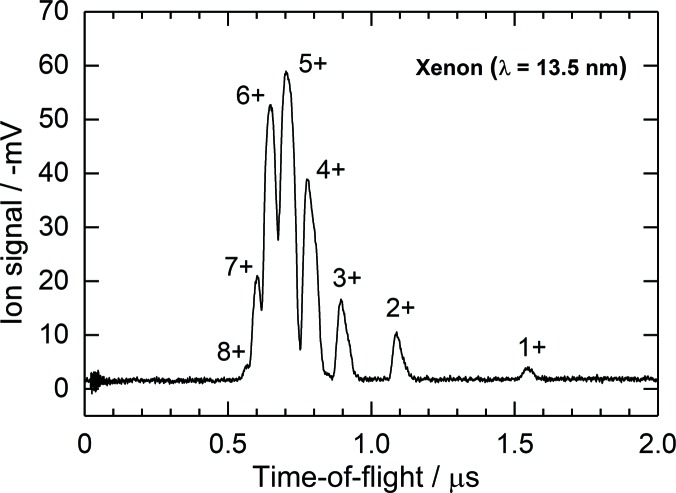
An example of an ion TOF spectrum of xenon obtained with the help of an XGMD. The data were obtained in the focus of the BL2 beamline at FLASH1 at a wavelength of 13.5 nm, with an average photon pulse energy of 6 µJ, a photon exposure of about 1 × 10^17^ cm^−2^ and an irradiance of about 2 × 10^13^ W cm^−2^.

The detection of fast photoelectrons allows single pulse-to-pulse read-out, *i.e.* pulse-resolved measurements. In-house-made high-voltage capacitors (0.8 nF) to separate the two parts of the electron split electrodes from the readout electronics were mounted in a vacuum directly on the electrode surface. This allows a significant improvement of the temporal resolution of less than 30 ns, as depicted in Fig. 4 ▸, depending on the bias voltage between the extraction and detection electrodes. The typical applied bias voltage is 200 V, which is high enough to suppress the emission of low-energy secondary electrons from the detection electrode. However, a number of high-energy elastically scattered secondary electrons can escape from the detection electrode and reach the interaction zone, where they are accelerated back towards the detection electrode. This process is repeated several times and stops after approximately 30 ns, as can be seen in Fig. 4 ▸ as small peaks arising after the main electron peak. The secondary electrons in fact lead to a signal broadening but do not affect the accuracy of the measurement because a given electron signal does not overlap with signals arising from the preceding and following photon pulses in the bunch train. The pulses in the bunch train are separated by 220 ns at the European XFEL (the trains are repeated with a frequency of 10 Hz), providing the highest repetition rate for FELs in the world. The electron signal is only used as a relative value and has the same shape at a particular photon energy and extraction voltage. When one changes the latter parameters this signal is cross-calibrated against the ion signal. Usually, we measure the amplitude and integral area of the main electron peak only after subtraction of the background. The ions are read out by a slow averaging ion-current measurement realized using a passive resistor–capacitor (RC) integrator with a time constant of 11 s which is not affected by any time structure of the radiation. Thus, ion-current measurements provide information about the average FEL pulse energy. As in the electron branch, a bias voltage of 200 V between the ion-detection electrode and the respective extraction electrode is applied in order to supress completely any secondary electron emission induced by ions hitting the detection electrode. The ion-detection electrode has a small rectangular aperture in the centre covered by an Ni grid with a length of 12 mm along the beam path. A fraction of ions pass through this aperture and are detected by a commercial open electron multiplier (ETP14880). Such a combination represents a compact ion time-of-flight (TOF) spectrometer with a moderate mass-to-charge resolution, as shown in Fig. 5 ▸. The measurement of ion TOF spectra may provide insight into the spectral purity of the FEL radiation, such as the contribution of high harmonics, and enable checking of the purity of the target gas used in the XGMD. Moreover, ion TOF spectra analysis enables us to deduce ion mean charge values in spectral ranges where no literature data are available (see *e.g.* Tiedtke *et al.*, 2014 ▸). The ion mean charge, together with the total photoionization cross section, are crucial fundamental data needed to determine the absolute photon flux by the XGMD.

Based on the Beer–Lambert law, the number of photons *N*_photon_ passing the detector is determined by the number of detected charge particles (ions or electrons) *N*_particle_,
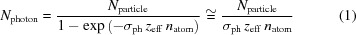
(for σ_ph_ *z*_eff_ *n*_atom_ << 1) where σ_ph_ is the total photoionization cross section, *z*_eff_ is the effective length along the photon beam path accepted by the respective electrode and *n*_atom_ is the density of the target gas atoms. This last is obtained according to *n*_atom_ = *p*/*kT* by determination of the gas pressure *p* using a calibrated spinning rotor gauge and the temperature *T* using a calibrated PT100 resistant thermometer, with the Boltzmann constant *k*. *N*_particle_ is determined by the charge *Q* accumulated by the respective electrodes according to *N*_particle_ = *Q*/*e*γ, where *e* is the elementary charge and γ is the mean charge of the photoions, which can be deduced from the measured ion TOF charge spectrum or taken from the literature (Suzuki & Saito, 1992 ▸). The total photoionization cross sections are well known from the literature (Henke *et al.*, 1993 ▸). For practical use, equation (1) for the average number of photons per pulse can be transformed to
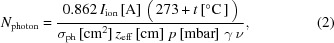
where *I*_ion_ and ν denote the total ion current from two split electrodes and the number of FEL pulses per second, respectively. In equations (1) and (2), the effective length *z*_eff_ is the quantity which has to be calibrated.

The XGMDs have been calibrated in different measurement campaigns over a period of six years at the MLS using monochromatic synchrotron radiation in the VUV spectral range (photon energies from 20 to 100 eV), *i.e.* in the regime of single and/or double and triple ionization with xenon or krypton as the target gas. Since the electron storage ring provides quasi-cw radiation with a repetition rate of 500 MHz in the microwatt regime, resulting in 10^10^ to 10^12^ photons per second, the XGMD could be absolutely calibrated only in the ion-current mode. The pulse-resolved electron signal is then cross-calibrated against the average absolute photon flux during XGMD operation at an FEL by simultaneously measuring the ion current and accumulating the corresponding number of electron pulses. The effective length for the ion detection is determined from equation (2) using the most accurate photoionization cross sections available (Samson & Stolte, 2002 ▸), and measuring the MLS photon flux with a calibrated photodiode (Gottwald *et al.*, 2010 ▸) and the total ion current by means of a calibrated electrometer. Table 1 ▸ summarizes the effective lengths obtained for different XGMDs constructed for three X-ray FELs. All data agree within the combined relative standard uncertainty of the order of 3.5%. The weighted average mean value of 22.16 cm agrees with the theoretical value of 22.20 cm, which is calculated taking into account the geometry of the ion split electrodes and the transmission of the Ni grid used at the extraction electrode. It should be mentioned that the relative standard uncertainty of the effective length amounts to 3.7%, which is consistent with the discrepancy between the data presented in Table 1 ▸. Table 2 ▸ summarizes all the contributions to the relative standard uncertainty of the effective length. The relative standard uncertainty of the absolute pulse energy of FEL radiation ranges between 5% and 8% depending on the spectral range. The main contributions to the latter uncertainty are the corresponding uncertainty of the effective length, the pressure, the temperature, the ion current (2% to 5%), the total photoionization cross section (2% to 5%) and the ion mean charge (2% to 3%). Further, despite the fact that calibration is carried out in the VUV range (a calibration of the XGMD in the X-ray range at synchrotron sources is not possible due to low photoionization cross sections and insufficient photon flux), the effective length can also be used in the hard X-ray range, subject to good agreement of the data obtained with the XGMD and the radiometer (Kato *et al.*, 2012 ▸; Song *et al.*, 2019 ▸).

**Figure 6:**
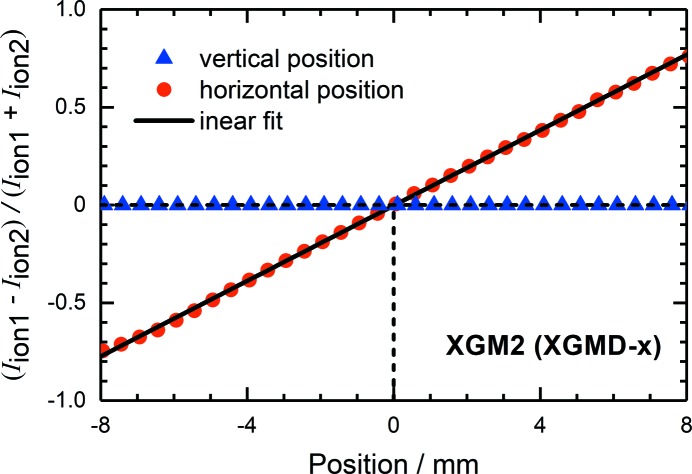
The fractional ratio of the two ion currents from a split electrode, together with a linear fit for the horizontally position-sensitive XGMD (X) of XGM2 as a function of the relative horizontal and vertical beam positions.

**Figure 7:**
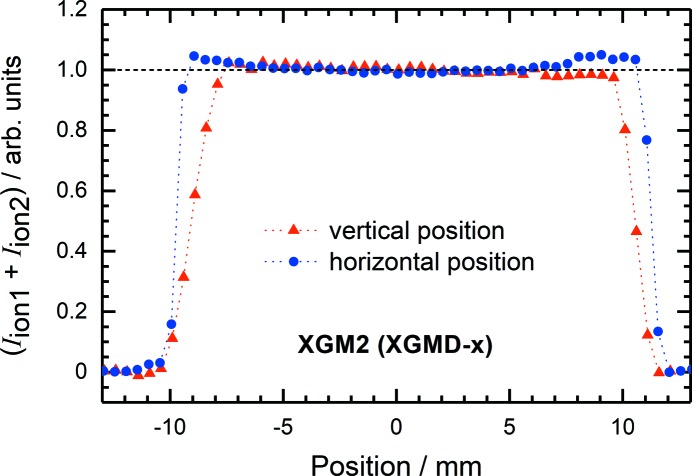
Total ion currents from a split electrode of XGMD (X) of XGM2 as a function of the relative beam position in the horizontal and vertical directions, demonstrating the detector’s spatial homogeneity.

While the total ion current provides information about the absolute photon flux, the ratio of the two split-electrode currents allows determination of the photon beam position. As an example, Fig. 6 ▸ shows the fractional ratio of two corresponding ion currents (*I*_ion1_ and *I*_ion2_) measured while moving the XGM girder horizontally and vertically with respect to the stable photon beam. For the XGMD (X), the data obtained for the horizontal position exhibit a linear behaviour but remain constant while moving the detector in the vertical direction. The linear fit of the experimental data represents a line with a slope of (0.0964 ± 0.0004) mm^−1^. From this, the accuracy of the beam-position monitoring is estimated and is of the order of 50 µm, which correlates with an ion-current difference of 1%. However, for ion currents higher than 10 pA, accuracies down to 10 µm may be achieved, depending on the read-out noise of the order of 0.02 pA. Finally, by measuring the total ion current for different beam positions as shown in Fig. 7 ▸, one may determine the active area and homogeneity of the detector, which are also important parameters for accurate absolute photon flux measurements. In both horizontal and vertical directions, the active area is about 20 mm, which is defined by the geometry of the detector. The present XGMD is installed behind a second one rotated by 90°. Thus, the size of the entrance aperture is 22 mm × 22 mm. A different behaviour of the signals around 10 mm and −10 mm is visible, and this can be explained by different photon beam sizes. In the horizontal direction the size is about 2 mm, while in the vertical direction the size is about 4 mm. However, by moving the detector by ±6 mm from the centre in both horizontal and vertical directions, the total ion current remains constant within ±1%.

### 2.2. Huge-aperture open electron multiplier (HAMP)   

**Figure 8:**
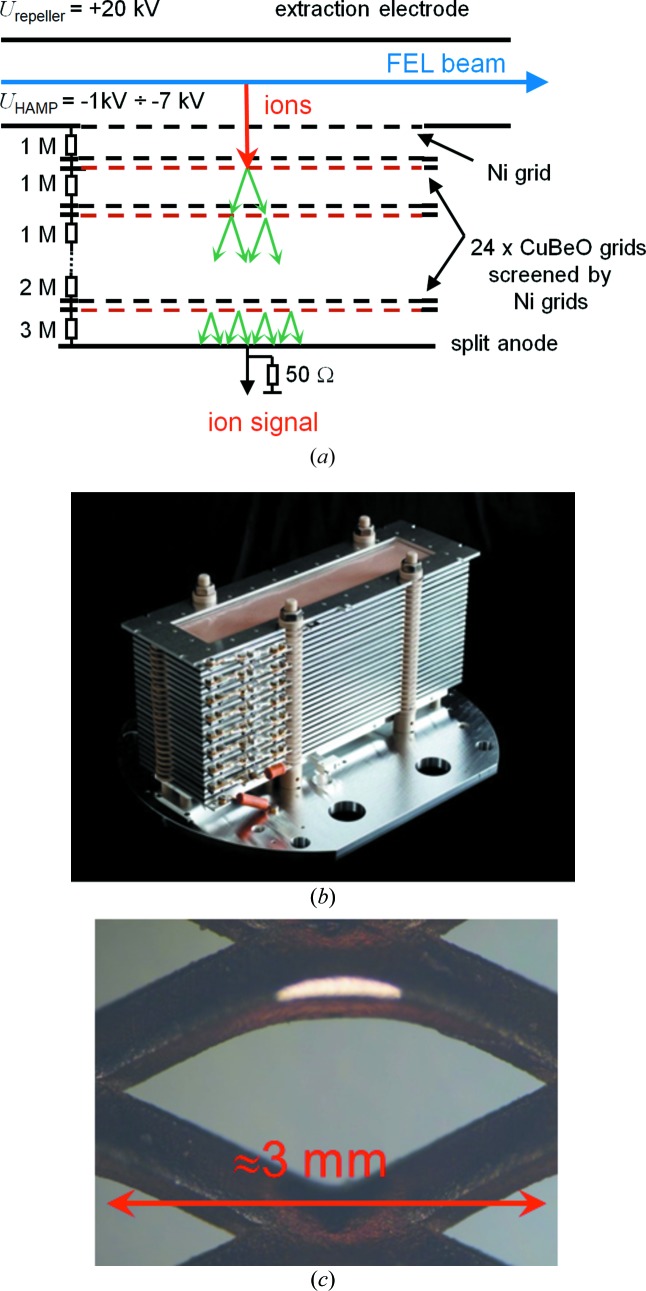
(*a*) Schematic diagram of the HAMP detector. (*b*) Picture of the HAMP detector. (*c*) Microscopic picture of the CuBeO grid structure, as used as dynodes for the HAMP.

An overview of the assembly and basic operation principle of the HAMP is shown in Fig. 8 ▸. The HAMP detector is an in-house-made multiplier with an active area as large as 200 mm along the photon beam path and 50 mm wide. Each HAMP is mounted on a DN300CF flange in a vacuum chamber, which is evacuated by the turbo-molecular pumps of the neighbouring XGMDs to a residual pressure of less than 10^−5^ Pa. The HAMP consists of 24 CuBeO grid dynodes previously activated at 873 K in an oven filled with CO_2_ at a pressure of 0.2 Pa. The transmission of the grids is 50% and their thickness is 0.5 mm. The distance between the grids amounts to 5 mm. A passive resistance divider of 29 MΩ connects the grids and allows the application of voltages of up to 7 kV in order to accelerate and multiply the secondary electrons created by ions. Ions generated upon photoionization are extracted from the interaction volume by a homogeneous electric field applied between a repeller electrode and the first grid, which are separated by 22 mm. The anode is realized by a triangular split electrode similar to the electron split electrodes in the XGMDs as shown in Fig. 3 ▸. Thus, the HAMP is capable of monitoring both the relative photon flux, which is cross-calibrated against the absolute value determined by the XGMD, and the photon beam position, as the grid structure of the HAMP keeps the information about the positions of ions hitting the first dynode, which in turn represents a projection of the FEL beam. The fractional current ratio from the two parts of the split electrode again represents a straight line, albeit with a smaller slope of 0.02 mm^−1^ compared with the XGMD detector. This difference is due to a broadening of the electron cloud while travelling through the grids. Therefore, the accuracy of the beam-position monitoring is also lower.

**Figure 9:**
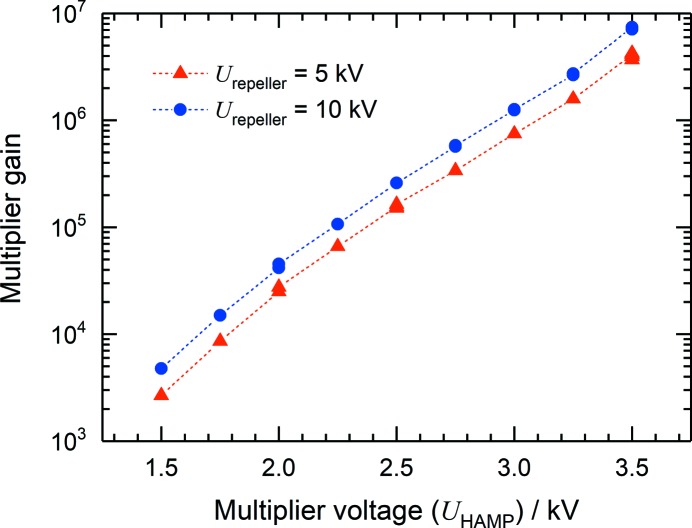
The typical gain of the HAMP detector as a function of the voltage applied to the divider. The data were obtained at the MLS for two different repeller voltages with krypton as the target gas at a wavelength of 40 nm, *i.e.* in the single photoionization regime.

Each CuBeO grid is screened by an Ni grid separated by 2 mm. The Ni grids have a transmission of 80% and are kept under the same potential as the associated CuBeO grid. Such a combination allows a significant increase in the gain of the multiplier, which is as high as 10^7^ even at a moderate voltage of 3.5 kV applied to the divider, as shown in Fig. 9 ▸. The gain was measured at the MLS by comparing the total current from the HAMP anode with the ion current from the XGMD, taking into account the different lengths of the detection electrodes. The data were obtained with krypton as the target gas at a wavelength of 40 nm, *i.e.* in the single photoionization regime. One should note that the gain rises with increasing kinetic energy of the ions, and hence depends on the potential at the repeller electrode as well as on the ion mass and charge state. Thus, the gain curves presented in Fig. 9 ▸ are typical examples for specific conditions, *i.e.* for repeller voltages of 5 and 10 kV and for singly charged krypton ions. In the X-ray spectral range where highly charged ions are generated, as well as for different repeller voltages and rare gases, the gain can be estimated assuming that the multiplier detection efficiency is proportional to the ion impact velocity (Schram *et al.*, 1966 ▸; Stockli & Fry, 1997 ▸). But in any case, the HAMP signal is a relative value as mentioned above, and any adjustment of the multiplier voltage and the respective gain must be such that the multiplier operates in a linear mode, as discussed below.

**Figure 10:**
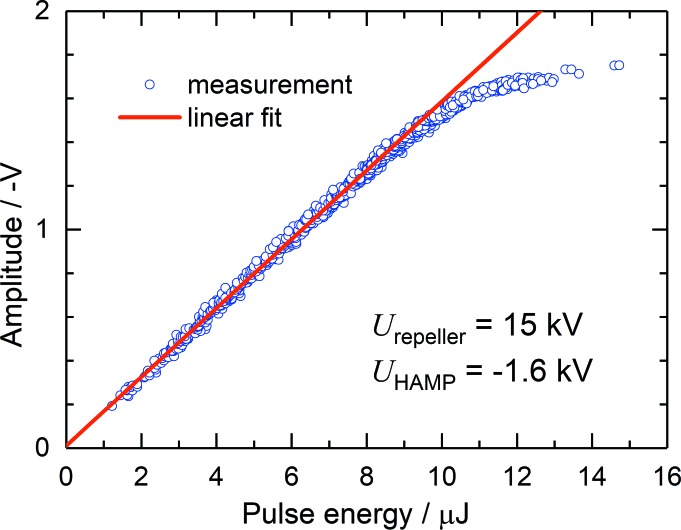
The amplitude of the pulse-resolved HAMP signal measured from one split electrode part as a function of the photon pulse energy. The red line represents a linear fit. The measurements were performed at FLASH 2 at a wavelength of 13.5 nm with xenon as the target gas.

HAMP allows pulse-resolved measurements as shown in Fig. 4 ▸. The signal presented there was measured at FLASH 2 with xenon at a wavelength of 13.5 nm by means of a digital oscilloscope. In this regime, singly, doubly and triply charged ions are created. Since their time of flight from the interaction volume to the first dynode is generally different, three pulses appear at the HAMP anode. However, by applying a high potential of up to 20 kV to the repeller electrode, the peaks arising from differently charged ions are compressed to one with a width of less than 200 ns, defining the temporal resolution of the HAMP detector. Finally, by simultaneously measuring the pulse energy with the help of the FLASH GMD, the linearity of the pulse-resolved HAMP signal could be demonstrated. The result is presented in Fig. 10 ▸. HAMP provides a fairly high linear response for pulse amplitudes of up to 1.5 V. These measurements are obtained in the single-bunch operation mode of FLASH with a repetition rate of 10 Hz. However, with increasing repetition rate one has to keep the amplitude lower because the total current of the HAMP detector should not exceed 500 nA. This current limit was determined at the MLS by varying the target gas pressure at different divider voltages and comparing the total current of the HAMP and XGMD.

## 3. Summary   

We have developed a novel X-ray gas monitor for free-electron lasers which represents an upgraded version of the gas-monitor detectors used at FLASH 1 from its first day of operation in 2005. A number of devices have now been constructed for permanent use at the European XFEL, the SwissFEL, LSLS II and FLASH 2. The X-ray gas monitor enables online monitoring of the absolute photon flux and photon beam position of FELs over a broad spectral range from VUV to hard X-rays. It covers a wide dynamic range from spontaneous undulator radiation to FEL radiation in the saturation regime with at least 10^15^ photons per pulse. The latter is the highest value which has been achieved at FELs so far. The detector provides a temporal resolution of better than 200 ns, a standard relative uncertainty for absolute photon pulse energy measurements of better than 10% and an accuracy of beam position measurements down to 10 µm.

## Figures and Tables

**Table 1:** Measured effective length of all existing XGMDs used in different devices (note that each XGM contains two XGMDs)

Detector	Effective length (cm)
XGM1 (Eur. XFEL)	22.13 ± 0.82
XGM2 (Eur. XFEL)	22.13 ± 0.82
XGM4 (Eur. XFEL)	21.24 ± 0.79
XGM5 (Eur. XFEL)	21.96 ± 0.81
XGM6 (Eur. XFEL)	22.71 ± 0.84
XGM7 (Eur. XFEL)	21.93 ± 0.81
XGM3 (SwissFEL)	22.75 ± 0.84
XGMD (LCLS II)	22.44 ± 0.83
XGMD (EUCALL)	22.28 ± 0.82
Mean value	22.16 ± 0.82

**Table 2:** Contributions to the relative standard uncertainty of the effective length

Source of uncertainty	Contribution to the relative standard uncertainty of the effective length (%)
Number of impact photons	
Photodiode spectral responsivity	2
Photodiode inhomogeneity	1.0
Photodiode current	0.2
Energy of the impact photons	0.2
Number of ions created	
Ion current	0.3
Ion current background correction	2.0
Atomic density measurements	
Target gas pressure	0.5
Temperature	0.2
Photoionization cross section data	2.0
Second order contribution	0.5
Total relative uncertainty (sum in quadrature)	3.7
